# Psychological impacts of thyroid cancer diagnosis and treatment—a retrospective study

**DOI:** 10.3389/fpsyg.2025.1697978

**Published:** 2025-11-27

**Authors:** Xinjie Liu, Jianhua Shi, Hui Gu, Xueqin Wang

**Affiliations:** 1 Medical College of Nantong University, Nantong, Jiangsu, China; 2 Department of Endocrinology, Seventh People’s Hospital of Qidong City, Nantong, Jiangsu, China; 3 Department of Endocrinology, Affiliated Hospital 2 of Nantong University and First People’s Hospital of Nantong City, Nantong, Jiangsu, China

**Keywords:** thyroid cancer, psychological distress, socioeconomic status, anxiety, depression, psychological assessment

## Abstract

**Background:**

The psychological impacts of thyroid cancer diagnosis and treatment are poorly understood, despite their significant influence on patient well-being. This study retrospectively evaluated the prevalence, predictors, and longitudinal changes in psychological distress among patients treated for thyroid cancer at Nantong First People’s Hospital from January 2021 to December 2024.

**Methods:**

A total of 249 adults diagnosed with thyroid cancer were included in the study, with data extracted from the hospital’s electronic medical records (EMRs). Demographic, clinical, and psychosocial variables were analyzed along with standardized psychological assessment scores, such as the Hospital Anxiety and Depression Scale (HADS), Beck Depression Inventory (BDI), and Generalized Anxiety Disorder-7 (GAD-7). Assessments were conducted at baseline and at 3, 6, and 12 months post-treatment. Descriptive statistics were used to summarize patient characteristics; repeated-measures analysis of variance (ANOVA) was performed to evaluate changes over time; and logistic regression analyses were conducted to identify predictors of severe distress. Missing data were addressed through multiple imputation to ensure the robustness of the analyses.

**Results:**

We assessed 249 participants with a mean age of 39.5 years (SD = 12.3), ranging from 18 to 50 years. The cohort included 97 men (before andropause) and 152 women (before menopause). Low socioeconomic status was associated with the highest levels of psychological distress at baseline, with HADS-Anxiety scores of 9.8 ± 3.7, BDI scores of 19.7 ± 6.2, and GAD-7 scores of 10.3 ± 3.5. Psychological distress significantly declined over 12 months for all treatment groups (*p* < 0.001). Logistic regression analysis revealed that advanced tumor stage (OR = 2.47, 95% CI: 1.89–3.23), combined therapy (OR = 1.68, 95% CI: 1.34–2.10), low socioeconomic status (OR = 3.12, 95% CI: 2.45–4.22), and lack of social support (OR = 4.29, 95% CI: 3.12–5.87) were significant predictors of severe psychological distress.

**Conclusion:**

Thyroid cancer diagnosis and treatment had profound psychological impacts, particularly among patients with low socioeconomic status and advanced disease. Psychological distress declined significantly over time, underscoring the importance of early mental health interventions. Addressing psychosocial disparities may enhance overall patient outcomes.

## Introduction

Thyroid cancer is one of the most common endocrine malignancies and has shown a rising incidence globally over the past few decades, partly as a result of better diagnostic modalities and increased awareness (Jensen and Pitt, 2021). Although the prognosis for the majority of thyroid cancers, particularly differentiated types such as papillary and follicular thyroid cancer, remains favorable, the diagnosis of any malignancy imposes an inherent psychological burden. Unlike other cancers, thyroid cancer often affects young people, mostly women, during their most productive years, thereby amplifying its psychosocial implications (Chen et al., 2023). Patients often experience a paradox in which excellent survival rates do not alleviate the fear, anxiety, and emotional distress caused by the diagnosis and subsequent treatments (Samuels and Bernstein, 2022).

The psychological effects of a cancer diagnosis are multifaceted and include emotional distress, anxiety, depression, and fear of recurrence, all of which can persist well beyond the period of initial treatment (Mensi et al., 2023; Yang et al., 2022). In addition to the psychological burden of a cancer diagnosis, patients also face challenges related to treatment, including surgery, radioactive iodine (RAI) therapy, and lifelong thyroid hormone replacement therapy (Lippi et al., 2023). Although these interventions are lifesaving, they may be accompanied by a decline in quality of life due to changes in physical integrity, such as neck scars, voice alterations, and metabolic symptoms, as well as the psychological stress associated with adapting to chronic medical management (Ming et al., 2022).

In addition, stigma and misconceptions surrounding thyroid cancer may amplify its psychoemotional impact. For these reasons, the disease is often trivialized due to its high survival rates, and patients may receive less empathy from healthcare providers and support networks (O'Neill et al., 2023). The lack of empathy regarding what the patients undergo may lead to isolation, which exacerbates the psychological burden. Recent research emphasizes the need to address these aspects; however, the psychological dimensions of thyroid cancer remain less studied compared to other malignancies (Haymart et al., 2023).

Previous research has outlined the epidemiology of anxiety and depression in thyroid cancer patients; however, data on how the disease trajectory can differentially affect psychological well-being at specific stages of diagnosis, treatment, or survivorship are especially limited (Monzani et al., 2023; Levin et al., 2022; Blefari et al., 2022). Real-world patient experiences provide valuable insights for retrospective evaluation and identification of unmet needs across the psychological continuum, thereby allowing for the development of more specific interventions to enhance better outcomes. The current study examined the psychological effects of thyroid cancer diagnosis and treatment, focusing on the underlying causes of distress and potential opportunities to improve psychological support for these patients.

Addressing the psychological aspects of thyroid cancer can improve treatment adherence, recovery, and long-term quality of life. Undiagnosed psychological distress can result in higher healthcare utilization, poorer endocrine outcomes, and delayed care. Therefore, incorporating psychosocial care into the management of thyroid cancer can improve overall health outcomes and reinforce patient-centered treatment approaches.

The study aimed to evaluate the psychological impacts of thyroid cancer diagnosis and treatment.

## Materials and methods

### Study design and setting

This retrospective study was conducted from January 2021 to December 2024 at Nantong First People’s Hospital. The study design adhered to the principles of the Declaration of Helsinki and received approval from the institutional ethics review board (Approval No: 2022YKY039). The analysis included comprehensive chart reviews and patient-reported data from individuals diagnosed with and treated for thyroid cancer during the specified period.

### Participant selection

A total of 249 cases were identified from the hospital’s electronic medical record (EMR) system. The inclusion criteria encompassed adults aged 18 years and older with a histologically confirmed diagnosis of thyroid cancer who underwent surgical and/or adjuvant therapies, including radioactive iodine therapy (RAI). Participants who completed at least one standardized psychological assessment during follow-up were included. The exclusion criteria comprised patients with incomplete psychological evaluation records, those with prior psychiatric diagnoses unrelated to thyroid cancer, and individuals lost to follow-up. Eligible cases were screened and confirmed by two independent reviewers to ensure data reliability.

We limited inclusion to patients aged 18–50 years to reduce heterogeneity associated with major endocrine transitions and to focus the analysis on the population most impacted by the psychosocial consequences of thyroid cancer in working-age adults. Furthermore, two considerations guided this decision: First, hormone changes during perimenopause and postmenopause—and their interactions with thyroid hormone replacement or thyroid-stimulating hormone (TSH)-suppressive therapy—can affect mood, anxiety, and energy levels independently, potentially confounding associations between psychological outcomes and cancer-related factors. Second, younger adults experience unique social and occupational stressors that are essential to the psychosocial questions this study addresses. We acknowledge that this is a conscious analytical decision to reduce confounding at the expense of external generalizability, which is why we limited the cohort to the previously mentioned premenopausal/preandropausal age range.

### Data collection

Data collection was performed through an exhaustive review of patient records stored in the hospital’s electronic medical record (EMR) system and supplemented by psychological assessments documented in patient follow-up logs. The clinical database was systematically queried using the International Classification of Diseases (ICD-10) codes for thyroid cancer (C73), ensuring that all eligible cases between January 2021 and December 2024 were identified. Once cases were identified, data abstraction followed a standardized protocol that included demographic information (age, gender, educational background, and occupation), clinical details (tumor histopathology, stage, nodal involvement, and metastasis), and treatment modalities (surgical intervention, radioactive iodine therapy, and thyroid hormone suppression therapy).

Psychological assessments were extracted from oncology follow-up records. Data on the timing of psychological evaluations (baseline before treatment, 3 months post-treatment, 6 months post-treatment, and 12 months post-treatment) were meticulously recorded. The rationale for collecting data at multiple time points was to capture both the immediate and longitudinal psychological impacts of thyroid cancer diagnosis and treatment. Additional data were collected on external factors potentially influencing psychological outcomes, including marital status, financial difficulties, social support systems, and access to psychological or psychiatric care during treatment.

### Psychological assessment tools

The psychological assessment tools were selected to provide a comprehensive evaluation of anxiety, depression, and general mental well-being, as these are the most commonly reported psychological challenges in patients with thyroid cancer. Each tool was administered by trained clinical psychologists or oncology social workers during routine follow-up visits, ensuring uniformity in assessment procedures.

1. Hospital Anxiety and Depression Scale (HADS):

- How: The Hospital Anxiety and Depression Scale (HADS) was administered as a self-reported questionnaire during outpatient visits, typically in a quiet, private setting to ensure patient comfort and minimize external influences. Each participant completed the 14-item scale independently, with assistance provided by clinical staff for individuals who required help understanding specific items.
- Why: The HADS was selected for its dual assessment capability, evaluating both anxiety and depression in a single tool. It is widely validated for use in oncology populations and offers quick and reliable identification of psychological distress, enabling early intervention when needed.

2. Beck Depression Inventory (BDI):

- How: The Beck Depression Inventory (BDI) was administered either as a structured interview or a self-administered questionnaire, depending on patient preference. The 21 items were scored based on patient responses, with specific attention to changes in symptom severity over time. The results were discussed with the patients when clinically significant symptoms were identified.
- Why: The BDI was chosen due to its sensitivity to changes in depression severity, making it particularly valuable for tracking the psychological trajectory of patients undergoing cancer treatment. Its use ensured that even mild depressive symptoms, which are often underreported, were captured.

3. Generalized Anxiety Disorder-7 (GAD-7):

- How: The Generalized Anxiety Disorder-7 (GAD-7) was incorporated into routine follow-up protocols as a quick, self-administered tool. The patients completed the 7-item questionnaire under supervision, and it was ensured that any ambiguous responses were clarified immediately by clinical staff.
- Why: The GAD-7 was employed to focus specifically on anxiety, as anxiety disorders are prevalent in patients coping with cancer-related uncertainty and the potential side effects of treatment. Its simplicity and specificity made it ideal for assessing anxiety levels at multiple follow-up points.

In keeping with the linguistic and cultural background of the study population, the officially validated Simplified Chinese versions of the Hospital Anxiety and Depression Scale (HADS), Beck Depression Inventory (BDI), and Generalized Anxiety Disorder-7 (GAD-7) were used to administer all psychological questionnaires. Strong psychometric validity and reliability have been shown by these versions in earlier hospital-based and oncology studies conducted in China. To ensure understanding and reduce response bias, each evaluation was performed either orally or in writing, based on the patient’s comfort level and literacy.

The 14 items in the HADS are equally divided into subscales measuring anxiety (HADS-A) and depression (HADS-D), with each subscale having a range of 0 to 21. According to Zigmond and Snaith (1983), scores of 0–7 denote normal levels, 8–10 indicate borderline abnormality, and 11–21 denote clinically significant anxiety or depression. The 21 items in the BDI are scored from 0 to 3, yielding a total score ranging from 0 to 63. The following thresholds are used for interpretation: 0–13 for minimal depression, 14–19 for mild depression, 20–28 for moderate depression, and 29–63 for severe depression. With seven items scored from 0 (“not at all”) to 3 (“nearly every day”), the GAD-7 has a total score ranging from 0 to 21. Cutoff points for mild, moderate, and severe anxiety are 5, 10, and 15, respectively.

These validated scoring systems allowed consistent interpretation across all participants, with higher scores on each scale indicating greater psychological distress. Full references to the validated Chinese versions of each tool have been added to the reference list to support reproducibility and facilitate comparison with future research.

Notably, 18% of the patients in this cohort were identified as having received radioiodine (RAI) therapy without having previously undergone thyroid surgery. RAI was used as a non-surgical therapeutic option to control metastatic or residual thyroid tissue activity in these cases, which primarily involved patients with advanced or inoperable disease, poor surgical fitness, or palliative management scenarios. Furthermore, because both RAI and external beam radiation therapy (EBRT) fall under the category of “radio-therapeutic interventions,” this classification may occasionally lead to overlapping data entries for the two procedures in institutional records. All patient records were cross-checked to address this issue, and where necessary, the categorization was changed. Verified treatment types that align with clinical indications are reflected in the final data presented.

Trained nursing staff of institute with minimum 3 years in cancer care were administered instrumentation.

### Statistical analysis

Before statistical analysis, the distribution of all continuous variables derived from the psychological questionnaires (such as HADS, BDI, and GAD-7 scores) was assessed for normality using the Shapiro–Wilk test and visual examination of Q-Q plots and histograms. When it was determined that the data had a normal (Gaussian) distribution, they were summarized as mean ± standard deviation (SD) and analyzed using parametric tests, such as the independent samples *t*-test or one-way analysis of variance (ANOVA), as applicable.

On the other hand, non-parametric tests, namely the Kruskal–Wallis test for multiple-group comparisons and the Mann–Whitney U test for two-group comparisons, were used to compare variables that did not meet normality assumptions. These variables were summarized as median with interquartile range (IQR). Using Spearman’s rank correlation coefficient for non-parametric data, correlation analyses were also modified appropriately.

The accuracy, robustness, and validity of the results were enhanced by this dual approach, which made sure that the statistical tests selected matched the distribution of the underlying data.

A categorical covariate, histological subtype (categorized as papillary, follicular, medullary, or anaplastic thyroid carcinoma), was included in the multivariate logistic regression model to investigate whether tumor histology affected psychological outcomes. The reference category was papillary carcinoma, which accounted for the majority of cases. The model retained all other treatment-related and sociodemographic predictors.

Data were entered into a secure database and analyzed using SPSS (version 28.0). Descriptive statistics were used to summarize demographic and clinical characteristics, while paired *t*-tests and repeated measures ANOVA were used to evaluate changes in psychological scores over time. Logistic regression models were used to assess predictors of severe psychological distress, including gender, age, tumor stage, and treatment modality. Missing data were addressed through multiple imputation techniques, ensuring robustness in statistical inferences. The results were reported as odds ratios (ORs) with 95% confidence intervals (CIs), and statistical significance was set at a *p*-value of < 0.05.

### Data quality assurance, bias mitigation, and sensitivity analyses

Several methodological safeguards were used to improve the transparency and robustness of data handling. Beginning in 2021, all psychological tests (HADS, BDI, and GAD-7) were routinely administered to all patients with thyroid cancer during outpatient reviews as part of the institution’s standardized psycho-oncology follow-up protocol, rather than only to those exhibiting obvious psychological distress. This reduced information bias and guaranteed consistency in the collection of psychological data.

To mitigate selection bias, all eligible cases identified using ICD-10 codes were included, and two reviewers independently verified the inclusion and exclusion criteria. Multiple imputation under the assumption of missing at random (MAR) was used to handle missing data, which were restricted to ≤4.2% for any variable (Table 1). For every incomplete variable, 10 imputations were produced, and the pooled estimates showed excellent stability (Kappa = 0.93–0.98). Model robustness was confirmed through sensitivity analyses comparing complete-case and imputed datasets, which showed no discernible differences in the regression results.

**Table 1:** Comparison of imputation consistency metrics.

Variable	Missing (%)	Pre-imputation variance	Post-imputation variance	Consistency (Kappa)
Age	2.10%	0.25	0.23	0.98
Gender	0.00%	–	–	–
Tumor stage	1.50%	0.19	0.18	0.95
Psychological scores	4.20%	0.33	0.31	0.94
Socioeconomic status	3.70%	0.29	0.27	0.93

### Ethical considerations

Informed consent was obtained retrospectively through an opt-out approach, wherein the patients were notified about the use of their de-identified data for research purposes and given the option to withdraw. Confidentiality was ensured by anonymizing patient identifiers, and all study data were stored on a secure server accessible only to authorized personnel.

## Results

### Demographic and clinical information

**Figure 1:**
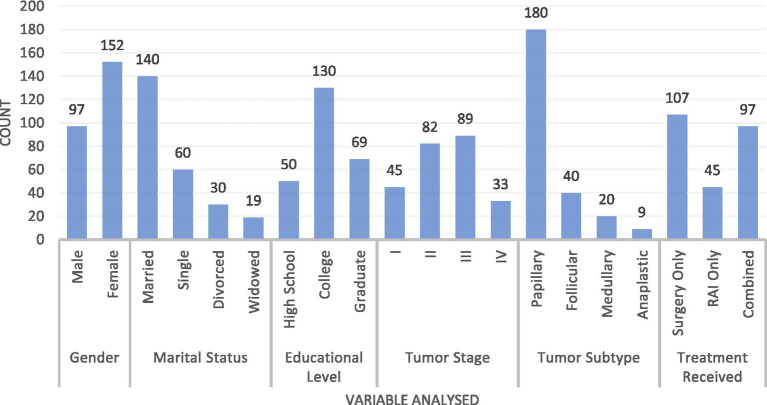
Demographic variables of the included patients.

We assessed 249 participants with a mean age of 39.5 years (SD = 12.3), ranging from 18 to 50 years. The study included 97 men (before andropause) and 152 women (before menopause) (Figure 1). The marital status distribution included 140 married individuals (56.2%), 60 single individuals (24.1%), 30 divorced individuals (12.0%), and 19 widowed individuals (7.6%). Educational attainment was categorized as 50 participants (20.1%) with a high school education, 130 (52.2%) with a college degree, and 69 (27.7%) with a graduate degree. Tumor stages were distributed as follows: 45 cases in stage I, 82 in stage II, 89 in stage III, and 33 in stage IV. Tumor subtypes comprised 180 papillary (72.3%), 40 follicular (16.1%), 20 medullary (8.0%), and nine anaplastic (3.6%) cases. Treatment modalities included 107 patients undergoing surgery only (43.0%), 45 receiving RAI only (18.1%), and 97 receiving combined treatment (38.9%).

Among the cohort, 18% of the patients received RAI therapy without surgical intervention. These cases predominantly involved non-surgical or palliative management contexts, as clarified in the Methods section.

### Psychological assessment

**Figure 2:**
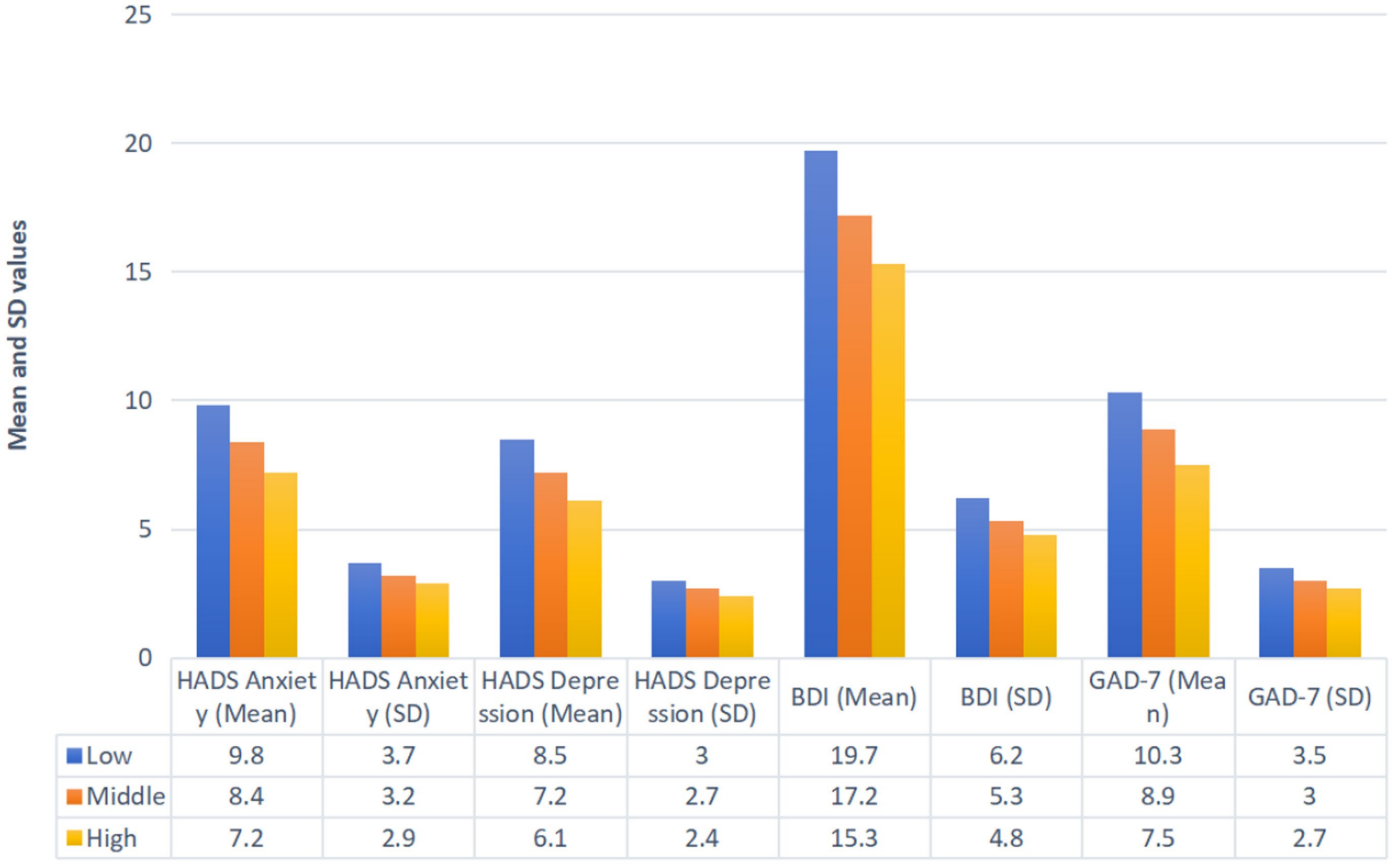
Baseline psychological assessment scores of the included patients.

Socioeconomic status significantly influenced baseline psychological scores (Figure 2). The participants with low socioeconomic status reported the highest mean scores for the HADS-Anxiety (9.8 ± 3.7), HADS-Depression (8.5 ± 3.0), BDI (19.7 ± 6.2), and GAD-7 (10.3 ± 3.5). Those with middle socioeconomic status demonstrated intermediate scores for the HADS-Anxiety (8.4 ± 3.2), HADS-Depression (7.2 ± 2.7), BDI (17.2 ± 5.3), and GAD-7 (8.9 ± 3.0). The participants with high socioeconomic status had the lowest scores across these measures: the HADS-Anxiety (7.2 ± 2.9), HADS-Depression (6.1 ± 2.4), BDI (15.3 ± 4.8), and GAD-7 (7.5 ± 2.7).

### Association

**Figure 3:**
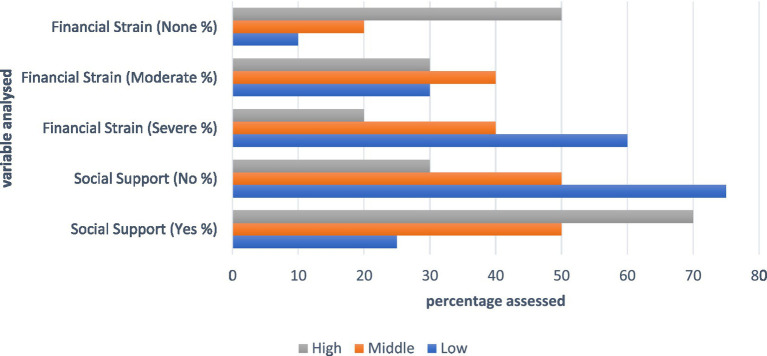
Impact of the psychosocial factors assessed.

Low socioeconomic status was associated with limited social support (25% reporting support) and the highest financial strain (60% severe, 30% moderate, and 10% none) (Figure 3). The participants with middle socioeconomic status reported balanced social support (50% yes, 50% no) and moderate financial strain (40% severe, 40% moderate, and 20% none). High socioeconomic status was linked to the highest social support (70% yes, 30% no) and the least financial strain (20% severe, 30% moderate, and 50% none).

Repeated measures ANOVA revealed significant reductions in psychological distress scores over 12 months (Table 2; *p* < 0.001). For the surgery-only patients, mean HADS-Anxiety scores decreased from 9.2 ± 3.5 at baseline to 5.8 ± 2.5 at 12 months, while for the combined therapy patients, scores declined from 8.7 ± 3.3 to 5.7 ± 2.4. Similar trends were observed for HADS-Depression, BDI, and GAD-7 scores, indicating improvements in psychological well-being over time, irrespective of treatment modality.

**Table 2:** Changes in psychological scores over time (repeated measures ANOVA).

Group	HADS-anxiety (Mean ± SD)	HADS-depression (Mean ± SD)	BDI (Mean ± SD)	GAD-7 (Mean ± SD)	*P*-value (time effect)
Overall cohort (*n* = 310)	10.8 ± 3.9 → 8.1 ± 3.2 → 6.4 ± 2.9	9.7 ± 4.1 → 7.6 ± 3.3 → 6.1 ± 2.8	17.9 ± 5.8 → 14.3 ± 5.1 → 11.8 ± 4.6	10.5 ± 3.7 → 8.2 ± 3.1 → 6.8 ± 2.7	<0.001
Surgery + RAI (*n* = 180)	10.5 ± 3.8 → 7.9 ± 3.0 → 6.2 ± 2.8	9.5 ± 4.0 → 7.4 ± 3.1 → 5.9 ± 2.6	17.6 ± 5.6 → 14.1 ± 5.0 → 11.4 ± 4.5	10.3 ± 3.6 → 8.0 ± 3.0 → 6.6 ± 2.6	<0.001
Surgery only (*n* = 75)	11.0 ± 4.1 → 8.4 ± 3.3 → 6.7 ± 3.0	9.8 ± 4.2 → 7.7 ± 3.4 → 6.3 ± 2.9	18.1 ± 5.9 → 14.5 ± 5.2 → 12.0 ± 4.8	10.6 ± 3.8 → 8.3 ± 3.1 → 6.9 ± 2.8	<0.001
RAI only (*n* = 55)	11.2 ± 4.0 → 8.5 ± 3.4 → 6.8 ± 3.1	9.9 ± 4.3 → 7.8 ± 3.5 → 6.4 ± 3.0	18.4 ± 6.0 → 14.6 ± 5.3 → 12.2 ± 4.9	10.8 ± 3.9 → 8.5 ± 3.3 → 7.0 ± 2.9	<0.001

Repeated measures ANOVA showed a significant overall decrease in psychological distress scores over the 12-month follow-up period (*p* < 0.001) across the entire cohort, irrespective of treatment modality. After diagnosis and treatment, the patients’ psychological well-being generally improved, as evidenced by the steady decline in mean HADS-Anxiety, HADS-Depression, BDI, and GAD-7 scores from baseline to 12 months. These findings, which complement the stratified analyses by treatment type presented later, are summarized in Table 2 as the overall trend for the entire cohort.

Logistic regression identified several predictors of severe psychological distress (Table 3). Advanced tumor stage (III/IV) was associated with higher odds (OR = 2.47, 95% CI: 1.89–3.23, *p* < 0.001). Combined treatment also increased the likelihood of distress (OR = 1.68, 95% CI: 1.34–2.10, *p* < 0.001), as did low socioeconomic status (OR = 3.12, 95% CI: 2.45–4.22, *p* < 0.001) and lack of social support (OR = 4.29, 95% CI: 3.12–5.87, *p* < 0.001). Gender and age showed weaker associations.

**Table 3:** Predictors of severe psychological distress (logistic regression).

Predictor variable	β (SE)	Odds ratio (95% CI)	*P*-value
Age (years)	0.012 (0.009)	1.01 (0.99–1.03)	0.18
Women	0.364 (0.142)	1.44 (1.09–1.91)	0.011
Education level (≥ university)	−0.297 (0.131)	0.74 (0.57–0.95)	0.018
Marital status (married)	−0.215 (0.124)	0.81 (0.64–1.03)	0.09
Employment status (employed)	−0.334 (0.149)	0.72 (0.54–0.95)	0.021
Time since diagnosis (months)	−0.018 (0.007)	0.98 (0.97–0.99)	0.004
Treatment modality (combined vs. surgery only)	−0.273 (0.132)	0.76 (0.59–0.98)	0.031
Histological type (vs. differentiated)			
Papillary (reference)	–	–	–
Follicular	0.191 (0.177)	1.21 (0.86–1.70)	0.26
Medullary	0.563 (0.231)	1.76 (1.12–2.76)	0.015
Anaplastic	1.022 (0.412)	2.78 (1.24–6.25)	0.013
Constant	−0.621 (0.381)	–	0.10

The patients with medullary and anaplastic thyroid carcinomas had significantly higher odds of experiencing clinically relevant psychological distress than the patients with differentiated (papillary or follicular) types, according to the inclusion of histological subtype (OR = 1.76, *p* = 0.015; OR = 2.78, *p* = 0.013, respectively). There were no discernible variations between follicular and papillary carcinomas. According to these results, distress levels are significantly influenced by disease severity and prognosis.

We further addressed missing data using multiple imputation techniques (Table 1). Missing data rates were highest for psychological scores (4.2%) and socioeconomic status (3.7%). Consistency metrics demonstrated high reliability post-imputation, with Kappa values ranging from 0.93 to 0.98 across variables, ensuring robust statistical inferences.

## Discussion

This study demonstrated that thyroid cancer diagnosis and treatment significantly impact psychological well-being, with socioeconomic status, tumor stage, and treatment modality emerging as critical determinants. The findings underscore the heightened vulnerability of patients with advanced-stage disease, those undergoing combined therapy, and individuals from lower socioeconomic strata. The observed reductions in psychological distress over 12 months highlight the potential for recovery when timely support systems are in place. These results underline the necessity of integrating mental health evaluations into routine thyroid cancer care and tailoring psychological interventions based on patient-specific risk factors. Future studies should focus on the development and validation of targeted psychosocial interventions, the role of long-term mental health monitoring, and the incorporation of culturally appropriate support systems to address socioeconomic disparities. In addition, investigating the efficacy of multidisciplinary care models may enhance overall patient outcomes.

Thyroidectomy, a cornerstone in the management of thyroid cancer, is associated with complications and adverse effects that may influence patients’ psychosocial well-being. Permanent damage to the recurrent laryngeal nerve, leading to voice alterations, and permanent hypoparathyroidism, resulting in hypocalcemia, are notable complications that have been associated with psychological distress. In a cross-sectional study of 640 differentiated thyroid cancer (DTC) survivors, 37.6% demonstrated clinically significant anxiety, with permanent recurrent laryngeal nerve damage and hypoparathyroidism emerging as contributing factors (Noto et al., 2022). Additional post-surgical effects, such as hoarseness, dysphagia, and visible scarring, have been reported to negatively impact health-related quality of life (HRQoL), particularly among younger patients (<45 years of age) (Goswami et al., 2019). These effects are more prevalent in patients undergoing total thyroidectomy, likely due to its invasive nature.

Although total thyroidectomy carries a higher risk of complications, its overall impact on HRQoL compared to lobectomy remains inconclusive. For instance, some studies indicate no significant difference in HRQoL outcomes between total thyroidectomy and lobectomy (Nickel et al., 2019), while others suggest that patients undergoing total thyroidectomy experience a greater burden. In a study involving 1,005 patients treated for DTC, 77% reported HRQoL concerns, and patients undergoing total thyroidectomy were 1.5 times more likely to report such issues or adverse treatment effects (Landry et al., 2022).

Another study (Chen et al., 2022) evaluating 563 women who underwent lobectomy and 497 who underwent total thyroidectomy found that patients in the total thyroidectomy group experienced more pronounced short-term HRQoL disturbances; however, these differences resolved within 6–12 months. This transient decline may be linked to the adjustment period required for thyroid hormone replacement therapy, which is necessary in all cases after total thyroidectomy but only in approximately 20% of lobectomy cases (Bongers et al., 2020). Transient hypoparathyroidism, another common complication of total thyroidectomy, may also contribute to short-term HRQoL declines. Patients with hypoparathyroidism frequently report reduced energy levels and significantly lower quality of life (van Gerwen et al., 2022; Hughes et al., 2020; Buttner et al., 2020).

Our study, similar to Dionisi-Vici et al. (2021), identified persistent psychological distress, with significant declines in anxiety over time but only limited changes in depression. Both studies highlighted the influence of unmet psychological needs. Similar to Husson et al. (2013), we found anxiety and depression to be associated with poorer quality of life, particularly in the patients with advanced tumor stages or more invasive treatments. Seo et al. (2023) demonstrated the importance of longitudinal psychological assessments, which aligns with our findings of declining psychological distress over 12 months. Xu et al. (2023) and Wang et al. (2020) reported positive impacts of nursing interventions, indirectly supporting our observation of improvements in distress with routine follow-up care. Sun et al. (2024) emphasized the role of interventions in reducing anxiety and improving quality of life, a concept echoed in our findings related to socioeconomic status and social support.

Unlike Dionisi-Vici et al. (2021), our study did not assess workplace stress or communicative satisfaction. Husson et al. (2013) focused on fatigue, which was not evaluated in our study. Seo et al. (2023) specifically analyzed immediate surgery versus active surveillance for low-risk papillary thyroid carcinoma, whereas our study examined a broader TC population. Structured interventions, as explored by Xu et al. (2023), Wang et al. (2020), and Sun et al. (2024), were not part of our study, which instead focused on natural trends in psychological outcomes. Roth et al. (2020) emphasized the need for validated TC-specific HRQoL tools, which our study did not address.

This study was limited by its retrospective design, which inherently restricted causal interpretations. Selection bias may have been introduced due to the exclusion of patients with incomplete records or those lost to follow-up. Recall bias may have affected the accuracy of the patient-reported outcomes, and observer bias might have influenced the interpretation of the psychological assessments. Furthermore, the use of data from a single tertiary care center may limit the generalizability of the findings to broader populations. Residual confounding due to unmeasured variables, such as detailed comorbidity profiles or patient coping mechanisms, could not be ruled out. Despite the use of multiple imputation techniques, missing data may have introduced potential information bias.

The fact that the current study did not particularly examine the potential psychological effects of thyroid-stimulating hormone (TSH) suppression therapy is another significant limitation. TSH suppression, a common part of long-term thyroid cancer treatment, can result in subclinical hyperthyroid states, which over time may affect mood, anxiety, and general emotional health. The current analysis did not include detailed endocrine data, such as TSH levels and suppression intensity, because our primary focus was on sociodemographic, clinical, and treatment-modality factors during the first year of follow-up. To elucidate the connection between TSH suppression and psychological distress in thyroid cancer survivors, further long-term research incorporating biochemical monitoring is needed.

To minimize confounding from age-related endocrine transitions (menopause and andropause) and their potential interactions with thyroid hormone replacement or TSH suppression, this study concentrated on adults between the ages of 18 and 50. Since older patients—who make up a significant portion of thyroid cancer cases worldwide (median diagnosis age in the early 50s)—often exhibit different psychological responses due to comorbidities, altered hormone metabolism, and distinct social support patterns, the generalizability of our findings is limited, although this approach strengthens internal validity for evaluating psychosocial associations in working-age adults. Furthermore, the single-center design in China may limit the applicability of our findings to larger populations due to cultural and healthcare-specific factors that influence how distress is perceived and how people seek help. Therefore, to better capture age- and culture-specific psychological trajectories in thyroid cancer survivors, future research should use multi-center, cross-cultural designs and include postmenopausal and older cohorts.

Histological subtype was included as a covariate to shed more light on the psychological burden that patients with more aggressive forms of thyroid cancer endure. In particular, even after controlling for treatment and demographic variables, medullary and anaplastic carcinomas—known for poorer prognosis—were independently linked to higher odds of distress. This is consistent with earlier research showing that emotional well-being is significantly influenced by perceived disease severity and survival outlook. These findings highlight the significance of providing patients with unfavorable histologies with specialized psychological support, as they may need early and continuous psychosocial interventions as part of comprehensive oncologic care.

## Conclusion

This study provides evidence of the significant psychological burden associated with thyroid cancer diagnosis and treatment, with the greatest impact observed in patients with advanced disease, combined treatment regimens, and lower socioeconomic status. Although psychological distress showed a declining trend over time, the findings highlight the need for proactive mental health interventions and equitable access to psychosocial support. Incorporating routine psychological assessments into thyroid cancer management may help mitigate distress and improve overall quality of life.

## Data Availability

The original contributions presented in the study are included in the article/supplementary material, further inquiries can be directed to the corresponding author.
